# Dietary nano-manganese supplementation enhances intestinal integrity, muscle traits, and tight junction protein expression in broilers

**DOI:** 10.14202/vetworld.2025.3135-3148

**Published:** 2025-10-26

**Authors:** Maha Saleem, Sajid Khan Tahir, Muhammad Shahbaz Yousaf, Muhammad Numan, Hafsa Zaneb, Habib Rehman

**Affiliations:** 1 Department of Physiology, University of Veterinary and Animal Sciences, Lahore 54000, Pakistan; 2 Quality Control Laboratory, Veterinary Research Institute, Lahore, Pakistan; 3 Department of Anatomy and Histology, University of Veterinary and Animal Sciences, Lahore 54000, Pakistan

**Keywords:** broiler performance, intestinal barrier, jejunal morphology, manganese nanoparticles, meat quality, tight junction proteins

## Abstract

**Background and Aim:**

Manganese (Mn) is an essential trace mineral for poultry, supporting skeletal development, metabolism, and intestinal health. Conventional inorganic Mn sources often have low bioavailability, leading to oversupplementation, environmental excretion, and mineral imbalance. Mn nanoparticles (Mn-NP) offer improved absorption and reduced environmental burden, but their graded effects on broiler growth, intestinal morphology, meat quality, and tight junction proteins remain underexplored. This study evaluated the impact of dietary Mn-NP supplementation on productive performance, serum metabolites, jejunal architecture, and intestinal barrier function in broilers.

**Materials and Methods:**

A total of 240-day-old broiler chicks were randomly assigned to six groups (n = 40; 4 replicates of 10 birds) and fed a basal diet (control), bulk Mn (80 mg/kg), or Mn-NP at 20, 40, 60, or 80 mg/kg for 35 days. Growth performance, visceral organ development, serum biochemistry, meat physicochemical attributes, jejunal morphology, and messenger RNA expression of claudin-5 (CLDN-5) and zonula occludens-1 (ZO-1) were evaluated.

**Results:**

Mn-NP supplementation did not significantly alter body weight or feed intake. However, the 40-Mn-NP group showed improved feed conversion ratio during weeks 2 and 3 compared with the 80-Mn-NP group. The gizzard weight decreased significantly at 40-Mn-NP, while bulk Mn increased cecal weight. Serum metabolites, including liver and kidney markers, remained unaffected across treatments, indicating no toxicity. Muscle pH45min was higher in 20- and 40-Mn-NP groups, while pH24 was reduced in the 40-Mn-NP and bulk Mn groups. Birds supplemented with 80-Mn-NP exhibited larger muscle fibers, whereas the 20-Mn-NP group showed higher fiber density. Jejunal villi were longer and crypts deeper in the 20-Mn-NP group, while tight junction proteins (CLDN-5, ZO-1) were significantly upregulated in the 60-Mn-NP group.

**Conclusion:**

Mn-NP supplementation at 40–60 mg/kg optimally enhanced feed efficiency, jejunal morphology, and intestinal barrier integrity without adverse health effects. These findings highlight Mn-NP as a sustainable alternative to conventional Mn supplementation, improving gut health and meat quality while reducing mineral excretion. Future studies should validate long-term safety and commercial-scale applications.

## INTRODUCTION

Trace minerals are commonly added to poultry diets in inorganic forms at varying levels to meet the nutritional demands of growing birds. However, their incomplete absorption leads to excessive excretion, contributing to environmental pollution. This limitation underscores the need for more efficient alternatives. Among these minerals, manganese (Mn) is indispensable for poultry, playing essential roles in growth, skeletal development, immunity, reproduction, and the metabolism of carbohydrates and fats [1–3]. Beyond these functions, Mn is also critical for maintaining intestinal mucosal integrity, sustaining balanced gut microbiota, and improving meat quality, making it a key component of poultry nutrition [4–7].

In recent years, broilers’ Mn requirements have nearly doubled [8], prompting the supplementation of higher levels in poultry diets [9, 10]. Deficiency of Mn results in poor growth, skeletal deformities, compromised immunity, and impaired intestinal barrier function [11, 12]. Conversely, excessive dietary Mn not only raises environmental concerns through increased excretion but also disrupts the bioavailability of other trace minerals such as copper, zinc, and iron [13, 14]. At present, both inorganic (e.g., oxides, sulfates, chlorides) and organic (e.g., chelates, propionates) Mn sources are used in poultry feed [13]. Conventionally, inorganic forms have been preferred due to their low cost, though they suffer from poor stability and bioavailability, along with anti-nutritional interactions such as phytate binding in the gut, which reduces absorption and increases excretion [15, 16]. Organic forms, despite being more expensive, demonstrate higher bioavailability, allowing lower inclusion rates to improve mineral deposition, antioxidant activity, and meat quality [16, 17].

Recently, nanotrace elements have emerged as promising alternatives in poultry nutrition. Their nanoscale dimensions confer unique properties, including greater membrane permeability, enhanced absorption, prolonged intestinal retention, and reduced fecal excretion [18–20]. Mn nanoparticles (Mn-NPs) differ from bulk Mn in their chemical, biological, and physical behavior [21], offering superior bioavailability due to their size-driven absorption efficiency [22]. Indeed, reduced Mn excretion in Mn-NP-fed birds compared with bulk Mn confirms their higher bioavailability [13]. Several studies highlight their benefits: improved ileal digestibility without growth impairment in turkeys [22] and enhanced growth performance and tibial strength in broilers supplemented with Mn-loaded chitosan nanoparticles [23]. However, not all findings are favorable; some reports indicate that Mn-NP inclusion may cause poor growth, hepatotoxicity, mitochondrial dysfunction, reduced villus height (VH), increased intestinal permeability, and downregulation of tight junction proteins [6].

Mechanistically, Mn-NPs may enhance Mn uptake in intestinal epithelial cells, as reflected in elevated Mn-superoxide dismutase (MnSOD) activity [1, 5, 24]. This activity reduces oxidative stress, preserves cellular integrity [25], and promotes the expression of tight junction proteins, including occludin, claudins (CLDN), and zonula occluden-1 (ZO-1). Improved antioxidant defenses and reduced cellular damage may also support healthier villus structure and crypt development, thereby strengthening jejunal morphology and intestinal barrier function [6]. Furthermore, Mn-driven upregulation of MnSOD plays a central role in sustaining intestinal barrier integrity [26].

Although Mn is well recognized as an essential trace mineral in poultry nutrition, most supplementation practices continue to rely on inorganic salts (sulfates, oxides, and chlorides) or, more recently, organic chelates and propionates. These forms suffer from limitations such as poor bioavailability, high excretion, and antagonistic interactions with other minerals, which raise concerns about both production efficiency and environmental sustainability. Emerging evidence highlights the potential of manganese nanoparticle (Mn-NP) to overcome these limitations by improving intestinal absorption, enhancing antioxidant activity, and reducing fecal mineral losses. However, the literature is fragmented and inconsistent. Some studies have demonstrated improved feed efficiency, tibial development, and intestinal morphology with Mn-NP supplementation, whereas others have reported adverse effects, including hepatotoxicity, oxidative stress, impaired growth, and downregulation of tight junction proteins. Furthermore, most existing studies have focused narrowly on growth performance or bone mineralization, while critical aspects such as meat quality, intestinal barrier integrity, and molecular regulation of tight junction proteins remain insufficiently explored. The graded effects of Mn-NP on broiler physiology, particularly in terms of intestinal microarchitecture, mucosal health, and meat physicochemical traits, are poorly characterized. This gap hinders the establishment of safe, effective, and sustainable dietary recommendations for Mn-NP use in commercial poultry production.

The present study was designed to provide a comprehensive evaluation of Mn-NP supplementation in broilers across multiple biological domains. Specifically, the study aimed to investigate the graded effects of Mn-NP on growth performance, visceral organ development, serum biochemical profiles, meat physicochemical attributes, jejunal morphology, and the expression of intestinal tight junction proteins. By directly comparing Mn-NP with conventional bulk Mn at different inclusion levels, this work sought to identify the optimal supplementation dose that supports productive performance, enhances gut integrity, and improves meat quality while minimizing adverse health effects and environmental excretion. Ultimately, the findings are expected to contribute to developing sustainable feeding strategies that leverage the advantages of nanotechnology to improve poultry health, welfare, and production efficiency.

## MATERIALS AND METHODS

### Ethical approval

This experiment was approved by the Institutional Ethical Review Committee, University of Veterinary and Animal Sciences, Lahore, Pakistan (Approval No. DR/439).

### Study period and location

The study was conducted from March 2022 to April 2022 at the animal shed area of the Department of Physiology, Lahore, Pakistan.

### Preparation and characterization of Mn-NP-loaded chitosan

**Figure 1:**
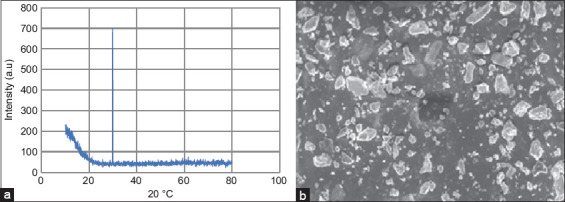
Characterization of nanoparticles: (a) X-ray diffraction graph showing the presence of the peaks of manganese (Mn) nanoparticles and (b) scanning electron microscopy was conducted to confirm the morphology and size of the Mn nanoparticles.

The Mn-NPs were prepared as previously described by Anwar [27]. Briefly, manganese dioxide (MnO_2_) nanoparticles were synthesized by the thermal decomposition of Mn (II) nitrate tetrahydrate at 350°C for 2 h. Subsequently, 1.5 g of the MnO_2_ nanoparticles was combined with 100 mL of 1.0% acetic acid (v/v) solution. The solution was thoroughly mixed for 1 h until it became clear after adding 0.4 g of chitosan. The pH was adjusted to 10 by adding 1.0 M NaOH. The obtained precipitate was subsequently heated for 5 h at 80°C and then crushed into powder form, resulting in the formation of chitosan loaded with Mn-NP. Characterization of Mn-NPs was performed using X-ray diffraction (XRD) and scanning electron microscopy (Figure 1). The XRD-based nanoparticle size was 24.87 nm.

### Experimental design and management of animals

A total of 240-day-old broiler chicks were individually weighed and randomly distributed (random number generation method) into six treatment groups (n = 40/group) with four replicate pens in a completely randomized design. Each replicate containing 10 birds was considered a pen. The birds were reared in an experimentally controlled house with wood shavings as bedding material under standard conditions. Each experimental pen measured 1 m^2^ and housed 10 birds (stock density: 10 birds/m^2^). Mechanical exhaust fans were used in combination with sidewall inlets to provide ventilation to control airflow. Continuous lighting (24 h light) was used for the entire experimental period. During the 1^st^ week of life, the chicks were kept at a room temperature of 35°C ± 2°C and a relative humidity of 65% ± 5%. The temperature was reduced by 3°C every week until it reached 26°C in the 3^rd^ week and then remained constant for the remainder of the experiment.

### Dietary and feeding treatments

The birds were fed with a corn-soybean-based basal diet containing 80 mg of Mn in the mineral mixture added to the ration, which was referred to as the control group. The other group, called the Mn-bulk group (Mn-Bulk), was fed the same basal diet supplemented with an additional 80 mg Mn/kg of diet in the form of MnSO_4_. The remaining groups received the basal diets supplemented with either 20 mg (20-Mn-NP), 40 mg (40-Mn-NP), 60 mg (60-Mn-NP), or 80 mg (80-Mn-NP) of Mn-NP/kg of diet for 35 days (Table 1). Water and feed were provided *ad libitum* during the experiment. The experimental diets were designed without the addition of any coccidiostats or antimicrobials and met or exceeded the National Research Council requirements [28]. The birds were vaccinated against Newcastle disease virus (Ceva-Phylaxia, Budapest, Hungary) on day 4 (intraocular; live attenuated) and day 20 (drinking water; live attenuated). The birds were also immunized against infectious bursal disease virus (Lohman Animal Health GmbH, Cuxhaven, Germany) on day 8 (intraocular; live intermediate strain) and day 24 (drinking water; live attenuated).

**Table 1:** Composition of the diets of the basal starter and growers.

Ingredients (g/kg)	Starter diet (1–21 days)	Grower diet (22–35 days)
Corn	576.8	605.3
Soybean meal	390.8	343.3
Soya oil	---	21.6
Di-calcium phosphate	8.0	5.7
Sodium chloride	3.7	3.6
Sodium bicarbonate	1.0	1.0
Lysine	2.6	2.9
Methionine	3.3	3.0
L-threonine	1.0	1.0
Choline chloride	1.0	1.0
Mineral premix	1.0	1.0
Vitamin premix	0.5	0.5
Lime	10.3	10.1
Total	1000	1000
Metabolizable energy (Kcal/kg)	2722.6	2890.6
Crude protein	23.08	21.16

Minerals per kg of the diet: Mg: 0.7 g, I: 1mg, Cu: 16 mg, Zn: 80 mg, Mn: 80 mg, Fe: 60 mg, Co: 0.4 mg, Se: 3 mg. Mn supplemented and measured concentration (mg/kg feed): Starter, 104 mg; Control, 181 mg; 20-Mn-NP, 124 mg; 40-Mn-NP, 143 mg; 60-Mn-NP, 164 mg; 80-Mn-NP, 183 mg; Grower, 102 mg; Bulk, 182 mg; 20-Mn-NP, 122 mg; 40-Mn-NP, 142 mg; 60-Mn-NP, 161 mg; 80-Mn-NP, 183 mg. *Supplied vitamins per kg of the diet: vitamin A: 11,000 IU; vitamin B12: 0.0532 mg, vitamin D3:2200 IU, vitamin E: 22 IU, riboflavin: 8.8 mg; pantothenic acid: 22 mg, ethoxyquin: 250 mg, menadione: 2.2 mg, pyridoxine: 4.4 mg; folic acid: 1.1 mg, biotin: 0.22, thiamine: 4.4 mg, Mn-NP = Manganese nanoparticles.

### Growth parameters

Weekly body weight (BW) and feed intake (FI) per pen were measured. Feed consumption was recorded by calculating the difference between the offered feed and the leftover feed. The feed conversion ratio (FCR) for each week was calculated by dividing the total feed consumed (g) per bird by the corresponding BW gain (g) for a particular week. The FCR was calculated per pen.

### Sample collection

On day 35, 12 birds per group (three birds/replicate) were randomly chosen and humanely euthanized through cervical dislocation for blood collection. Blood samples were collected from the jugular vein on day 35 using sterile syringes. Approximately 5–6 mL of blood was drawn into non-heparinized tubes and immediately processed for serum separation. To obtain serum, blood samples were centrifuged at 1,500 × *g* for 20 min at 4°C and subsequently stored at −20°C until analysis. Tissue samples were collected from the jejunum at the Meckel’s diverticulum junction for intestinal mucosal microarchitecture. For various gene expression in the jejunal tissue, jejunal tissues (size 1 × 1 × 1 cm) were collected approximately 1.0 cm before the Meckel’s diverticulum, rinsed with cold phosphate-buffered saline, and stored at −80°C for subsequent molecular analysis. Samples were removed and preserved for histology of pectoral muscles by immersing them in 10% buffered formalin.

### Determination of viscera weight

The sampled birds were eviscerated to collect various viscera. Digesta were removed from the gizzard, cecum, and small intestine; washed with ice-cold water; gently pressed and weighed. The relative weights of the liver, heart, gizzard, spleen, bursa, pancreas, small intestine, and cecum were calculated using the following formula:

Relative weight (%) = (Weight of the organ/Live body weight) × 100.

### Serum biochemical analysis

At the time of analysis, the serum samples were thoroughly vortexed after thawing. Subsequently, lipid profile (triglycerides [TG], total cholesterol [TC], and high-density lipoprotein [HDL]), renal markers (creatinine, uric acid, and blood urea nitrogen [BUN]), serum proteins (total protein [TP] and albumin), and liver markers (alanine aminotransferase [ALT] and aspartate aminotransferase [AST]) were measured using commercial kits (Dia Sys, Diagnostic System GmbH, Holzheim, Germany) as per manufacturer’s instructions using an Epoch plate reader (Biotek Instruments Inc., Winooski, USA). Globulin levels were determined by subtracting albumin from TP. The albumin-to-globulin ratio was calculated by dividing the serum albumin concentrations by globulin.

### Meat physicochemical traits

The pH of the pectoral muscle was determined using a pH meter (Model 228 M, PCE Instruments, Southampton, UK) with a probe electrode that was placed into muscle tissue, at least 1 cm deep, at 45 min, and 24 h post-slaughter [29].

To determine the cooking loss, approximately 100–150 g of pectoral muscle was collected, weighed (W1), and wrapped in inflated plastic bags to avoid evaporation. The samples were immersed in a water bath at 80°C for 1 h. Once the internal temperature of the plastic bags reached 78°C, they were gently blotted dry with paper towels without applying pressure and then cooled by submerging in running water for 30 min. After cooling, the muscle samples were removed from the polyethylene bags and reweighed (W2) to determine the cooking loss percentage using a previously described formula [30]:

Cooking loss (%) = (W1−W2)/W1 × 100.

The water-holding capacity was determined as previously described by Kudryashov and Kudryashova [31] with slight modifications. Briefly, 10 g of pectoral muscle was covered with Whatman No. 1 filter paper and placed in Falcon tubes (Corning, USA). Centrifugation was performed at 1,500 × *g* for 20 min, and the weight loss of the sample was measured. The water-holding capacity was calculated using the following formula:

Water-holding capacity (%) = (W1–W2)/W1 × 100.

### Muscle histomorphometry

For histomorphometry, pectoral muscle samples were micro-sectioned and stained with hematoxylin and eosin (H&E). Muscle histomorphometry assessment included the measurement of muscle fiber diameter (MFD), muscle surface area (MSA), and muscle fiber density (MFDe). Images of the slides were captured in five different zones on each slide and analyzed using the ImageJ software (https://imagej.net/ij/) to determine MFD. For muscle fiber counting, circles with a diameter of 0.22 mm were delineated on the images taken with a 10× objective lens. Each circle was split into two halves, and a specific counting rule was established to exclude muscle fibers in contact with the left side of the circle, thereby minimizing counting errors. The MFDe, measured as muscle fibers per square millimeter, was calculated using the following formula [32]:

Muscle fiber number/mm^2^ = (1/πr^2^) × number of fibers.

### Evaluation of jejunal morphology

Five μm thick jejunal tissue (three cross-sections from each sample) was cut using a microtome and fixed on glass slides. The jejunal tissues were stained using the H&E technique to assess various characteristics. For each sample, 15 intact and properly oriented crypt–villus units were randomly selected. The mean values obtained from each bird were used for statistical analysis. All measurements were performed under a microscope (Olympus AX70, Olympus Corp., Tokyo, Japan) fitted with a digital imaging system (Olympus DP20, Olympus USA). VH was defined as the length from the tip of the villus to the villus–crypt junction, whereas crypt depth (CD) was defined as the depth of the invagination between two consecutive villi. The villus surface area (VSA) was calculated using the following equation:

VSA (mm^2^) = (2π) × (VW/2) × (VH).

**Figure 2:**
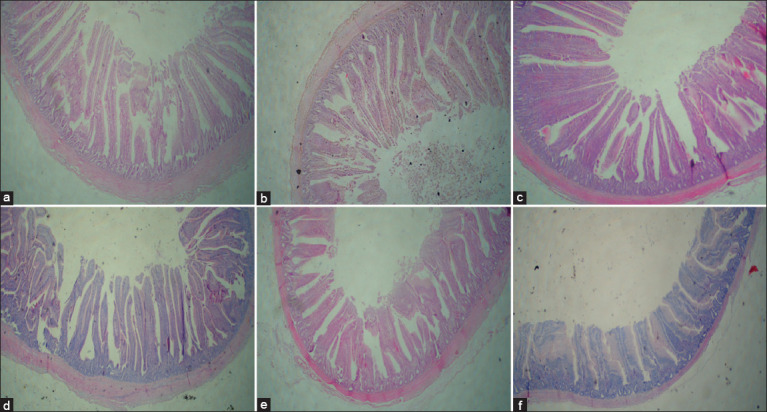
Photomicrographs of a hematoxylin and eosin-stained transversal section of jejunum morphology using a 4× objective lens. (a) Control = Basal diet without manganese (Mn) supplementation, (b) Bulk-Mn = Diet supplemented with Mn at 80 mg/kg of diet in the form of MnSO_4_, (c) 20-Mn-NP = 20 mg Mn-NP/kg of diet, (d) 40-Mn-NP = 40 mg Mn-NP/kg of diet, (e) 60-Mn-NP = 60 mg Mn-NP/kg of diet, and (f) 80-Mn-NP = 80 mg Mn-NP/kg of diet. Mn-NP = Manganese nanoparticles.

A single examiner who was blinded to the treatment groups performed all histological evaluations. Figure 2 shows the histological photographs.

### Analysis of gene expression in jejunal tissue

The messenger RNA (mRNA) was extracted from jejunal tissues using a magnetic bead method nucleic acid extraction system (HERO32, Ascend; Luoyang Ascend Biotechnology Co., Ltd, Henan, China) with the help of a nucleic acid extraction kit (Luoyang Ascend Biotechnology Co., Ltd). The concentration of extracted mRNA was determined using the NanoDrop (Thermo Fischer Scientific, Wilmington, DE, USA). The mRNA was reverse transcribed to generate complementary DNA (cDNA) using a cDNA synthesis kit (GeneDireX Inc., Taiwan) according to the manufacturer’s instructions.

The primer sequences used for CLDN-5 and ZO-1 quantification are shown in Table 2:

**Table 2:** Primer sequences used for real-time PCR.

Genes	Forward primer (5’- 3’)	Reverse primer (5’- 3’)	Annealing temperature (°C)
*CLDN-5*	CATCACTTCTCCTTCGTCAGC	GCACAAAGATCTCCCAGGTC	61°C
*ZO-1*	CTTCAGGTGTTTCTCTTCCTCCTC	CTGTGGTTTCATGGCTGGATC	62°C
*GAPDH*	CGATCTGAACTACATGGTTTACATGTT	CCCGTTCTCAGCCTTGACA	61°C

CLDN-5 = Claudin-5, ZO-1 = Zonula occluden-1, GAPDH = Glyceraldehyde 3-phosphate dehydrogenase, PCR = Polymerase chain reaction.

- CLDN-5 forward primer: 5′-CATCACTTCTCCTTCGTCAGC-3′
- CLDN-5 reverse primer: 5′-GCACAAAGATCTCCCAGGTC-3′
- ZO-1 forward primer: 5′-CTTCAGGTGTTTCTCTTCCTCCTC-3′
- ZO-1 reverse primer: 5′-CTGTGGTTTCATGGCTGGATC-3′.

Quantitative polymerase chain reaction (qPCR) was conducted using the Simply Green qPCR Master mix kit (GeneDireX Inc.). The reaction mixture included SYBR Green master mix, cDNA, and primers. The polymerase chain reaction program used to target mRNA amplification in samples was as follows: initial denaturation for 10 min at 95°C, followed by 45 cycles of 95°C for 15 s, 61°C–62°C for 30 s, and 72°C for 10 s. Relative mRNA expression levels were normalized by the housekeeping gene (glyceraldehyde-3-phosphate dehydrogenase). The relative quantities of mRNA were calculated by the 2^–ΔΔCt^ method [33].

### Statistical analysis

To determine whether the data were normally distributed, the Kolmogorov–Smirnov test was employed. Data homogeneity was assessed using Levene’s test (Statistical Package for the Social Sciences version 22.0, IBM, Armonk, NY, USA). Results are expressed as means and standard error of pooled means. For growth performance, the pen was considered the experimental unit, whereas individual birds were considered experimental units for measuring visceral development, health biomarkers, physicochemical variables of pectoral muscles, histomorphometry of pectoral muscles and intestinal mucosa, and gene expression. The effects of supplementation among the variables were analyzed using one-way analysis of variance. Tukey’s *post hoc* test was used to separate the means of the groups. The significance level was set at p < 0.05. Polynomial contrasts were used, if p < 0.05, to determine the linear and quadratic effects of the supplementation.

## RESULTS

### Growth performance

As presented in Table 3, supplementation with Mn-NP did not negatively influence growth performance. Weekly average BW and FI remained unaffected throughout the experimental period. No significant differences in FCR were observed among groups during weeks 1, 4, and 5. However, during week 2, birds receiving 20- and 40-Mn-NP exhibited significantly lower FCR (p < 0.05) compared with those supplemented with 80-Mn-NP, with a clear quadratic effect (p < 0.05). In week 3, the lowest FCR (p < 0.05) was recorded in the 40-Mn-NP group compared with the 80-Mn-NP group, accompanied by a significant linear effect (p < 0.05). No mortality or morbidity was observed during the trial.

**Table 3:** Effects of Mn-NP supplementation on broiler growth performance.

Parameters	Day	Control	Bulk-Mn (80 mg/kg)	Mn-NP doses (mg/kg)				SEM	p-value	Linear	Quadratic

				20	40	60	80				
BW (g)	7	191	197	190	194	193	195	1.10	0.468	0.591	0.961
	14	464	466	469	470	471	445	3.50	0.262	0.263	0.07
	21	851	844	869	845	877	819	9.70	0.615	0.229	0.182
	28	1261	1256	1261	1224	1392	1227	18.00	0.056	0.229	0.182
	35	1712	1729	1724	1708	1854	1704	23.00	0.420	0.428	0.616
FI (g)	7	164	166	166	168	167	165	1.72	0.993	0.812	0.565
	14	378	383	377	368	385	366	3.70	0.631	0.414	0.733
	21	573	573	572	554	597	577	7.00	0.698	0.799	0.552
	28	647	652	622	620	667	663	11.00	0.766	0.550	0.939
	35	965	1022	972	1003	1021	956	17.50	0.838	0.565	0.991
FCR	7	1.15	1.10	1.17	1.14	1.14	1.12	0.05	0.669	0.837	0.535
	14	1.38^ab^	1.43^ab^	1.35^b^	1.34^b^	1.38^ab^	1.46^a^	0.05	0.007	0.183	0.004
	21	1.51^abc^	1.55^ab^	1.43^bc^	1.39^c^	1.42^bc^	1.63^a^	0.02	0.007	0.05	0.460
	28	1.61	1.60	1.63	1.67	1.30	1.67	0.06	0.496	0.488	0.549
	35	2.15	2.17	2.11	2.08	2.27	2.01	0.05	0.723	0.508	0.354

Control = Basal diet without Mn supplementation, Bulk-Mn = Diet supplemented with Mn at 80 mg/kg of diet in the form of MnSO_4_, 20-Mn-NP = 20 mg Mn-NP/kg of diet, 40-Mn-NP = 40 mg Mn-NP/kg of diet, 60-Mn-NP = 60 mg Mn-NP/kg of diet, 80-Mn-NP = 80 mg Mn-NP/kg of diet, Mn-NP = Manganese nanoparticles, SEM = Standard error of pooled means, BW = Body weight, FI = Feed intake, FCR = Feed conversion ratio.

abc Means in a row lacking similar superscripts differ significantly at p < 0.05. Each group contained 40 birds distributed in 4 replicates with 10 birds per replicate. Values are means of 4 replicates.

### Relative viscera weights

The inclusion of Mn-NP did not significantly alter the relative weights of most visceral organs, with the exception of the gizzard and cecum (Table 4). Birds supplemented with 40-Mn-NP had a significantly reduced relative gizzard weight (p < 0.05) compared with the control group, showing a linear effect (p < 0.05). In contrast, cecal relative weight was significantly increased (p < 0.05) in the bulk-Mn group compared with the control, 60-Mn-NP, and 80-Mn-NP groups.

**Table 4:** Effects of Mn-NP supplementation on the relative visceral weights (%) of broilers.

Organs	Control	Bulk-Mn (80 mg/kg)	Mn-NP doses (mg/kg)				SEM	p-value	Linear	Quadratic

			20	40	60	80				
Liver	2.86	2.70	2.59	2.71	2.93	2.89	0.061	0.563	0.463	0.170
Heart	0.44	0.45	0.43	0.41	0.42	0.40	0.008	0.471	0.061	0.878
Gizzard	2.07^a^	1.93^ab^	1.95^ab^	1.61^b^	1.80^ab^	1.76^ab^	0.037	0.003	0.05	0.106
Spleen	0.14	0.12	0.14	0.12	0.10	0.10	0.006	0.131	0.025	0.367
Bursa	0.13	0.13	0.14	0.11	0.14	0.11	0.007	0.624	0.394	0.817
Pancreas	0.23	0.18	0.22	0.2	0.21	0.21	0.006	0.337	0.756	0.429
Small intestine	2.31	2.40	2.43	2.35	2.47	2.23	0.045	0.737	0.870	0.254
Cecum	0.14^b^	0.26^a^	0.17^ab^	0.17^ab^	0.14^b^	0.13^b^	0.052	0.009	0.052	0.099

ab Means in a row lacking similar superscripts differ significantly at p < 0.05. n = 12 birds/group (3 birds/replicate/group). Mn-NP = Manganese nanoparticles, SEM = Standard error of pooled means.

### Serum biochemical profile

Serum biochemical analysis indicated that Mn-NP supplementation did not induce toxicity. Health biomarkers, including TC, TG, HDL, ALT, AST, creatinine, uric acid, BUN, TP, albumin, globulin, and the albumin-to-globulin ratio, remained unaffected across all treatments compared with the control group (Table 5).

**Table 5:** Effects of Mn-NP supplementation on serum broiler metabolites.

Parameters	Control	Bulk-Mn (80 mg/kg)	Mn-NP doses (mg/kg)				SEM	p-value	Linear	Quadratic

			20	40	60	80				
TC (mg/dL)	111.18	115.76	112.32	118.31	120.52	113.47	1.85	0.703	0.443	0.368
TG (mg/dL)	105.06	112.98	99.49	101.91	109.71	120.32	2.22	0.063	0.094	0.056
HDL (mg/dL)	56.76	57.76	53.72	53.50	53.45	54.63	1.58	0.469	0.137	0.391
ALT (U/L)	1.24	2.19	1.74	1.74	1.70	2.32	0.26	0.884	0.523	0.925
AST (U/L)	33	32	38	34	38	26	2.16	0.652	0.650	0.232
Creatinine (mg/dL)	0.31	0.31	0.38	0.25	0.36	0.22	0.05	0.943	0.641	0.632
Uric acid (mg/dL)	7.80	7.67	8.46	7.47	7.62	7.63	0.10	0.112	0.361	0.444
BUN (mg/dL)	7.25	6.58	6.99	6.95	7.03	7.08	0.13	0.760	0.874	0.401
TP (g/dL)	3.92	3.82	3.76	3.55	4.16	3.20	0.10	0.082	0.118	0.386
Albumin (g/dL)	2.54	2.41	2.55	2.54	2.5	2.57	0.03	0.756	0.513	0.632
Globulins (g/dL)	1.38	1.40	1.20	1.01	1.66	0.63	0.11	0.055	0.083	0.316
The albumin/globulin ratio	2.11	1.79	2.20	3.91	2.30	2.44	0.27	0.363	0.389	0.408

Mn-NP = Manganese nanoparticles, TC = Total cholesterol, TG = Triglycerides, HDL = High-density lipoproteins, ALT = Alanine aminotransferase, AST = Aspartate aminotransferase, BUN = Blood urea nitrogen, TP = Total protein, SEM = Standard error of pooled means. n = 12 birds/group (3 birds/replicate/group).

### Meat physicochemical traits

Breast muscle pH measured at 45 min post-slaughter (pH 45 min) was significantly higher (p < 0.05) in the 20- and 40-Mn-NP groups compared with the 80-Mn-NP group (Table 6). In contrast, pH measured after 24 h (pH 24 h) was significantly lower (p < 0.05) in birds receiving bulk-Mn and 40-Mn-NP compared with 80-Mn-NP, exhibiting a quadratic effect (p < 0.05). Cooking loss and water-holding capacity of pectoral muscles were not influenced by Mn-NP supplementation (Table 6).

**Table 6:** Effects of Mn-NP supplementation on broiler meat physicochemical traits and histology.

Parameters	Control	Bulk-Mn (80 mg/kg)	Mn-NP doses (mg/kg)				SEM	p-value	Linear	Quadratic

			20	40	60	80				
pH_45 min_	6.13^ab^	6.16^ab^	6.33^a^	6.39^a^	6.15^ab^	5.94^b^	0.04	0.056	0.231	0.05
pH_24 h_	5.46^ab^	5.31^b^	5.39^ab^	5.29^b^	5.46^ab^	5.52^a^	0.02	0.05	0.068	0.05
WHC (%)	15.92	25.05	20.50	18.45	14.37	16.14	1.46	0.332	0.277	0.293
CL (%)	18.58	21.88	20.12	18.37	19.34	17.04	0.65	0.381	0.207	0.248
Histology										
MFD (µm)	37.13^b^	36.88^b^	37.02^b^	37.55^b^	42.22^ab^	44.81^a^	0.75	<0.05	<0.05	0.051
MSA (µm^2^)	1133^b^	1120^b^	1112^b^	1157^b^	1455^ab^	1640^a^	48.00	<0.05	<0.05	0.007
MFDe (cell/mm^2^)	261^ab^	222^bc^	301^a^	224^bc^	188^c^	210^bc^	8.54	<0.05	0.05	0.229

pH_45 min_ and pH_24 h_ represent pH of pectoral muscle measured at 45 min and 24 h post-slaughter, respectively, Mn-NP = Manganese nanoparticles, WHC = Water holding capacity, CL = Cooking loss, MFD = Muscle fiber diameter, MSA = Muscle fiber surface area, MFDe = Muscle fiber density, SEM = Standard error of pooled means. ^abc^Means in a row lacking similar superscripts differ significantly at p < 0.05. n = 12 birds/group (3 birds/replicate/group).

Muscle fiber characteristics were markedly affected. Birds supplemented with 80-Mn-NP had significantly greater MFD and surface area (p < 0.05) compared with the control, bulk-Mn, 20-Mn-NP, and 40-Mn-NP groups. Both linear and quadratic effects (p < 0.05) were observed for these parameters. Conversely, MFDe was significantly higher (p < 0.05) in the 20-Mn-NP group compared with Mn-supplemented groups, with a pronounced linear effect (p < 0.05).

### Jejunal morphology

Histomorphometric evaluation (Table 7) revealed significant changes in the architecture of the jejunum. VH was significantly reduced (p < 0.05) in the 80-Mn-NP group compared with other groups, with both linear and quadratic effects (p < 0.05). Villus width was significantly greater (p < 0.05) in the 40- and 60-Mn-NP groups compared with the bulk-Mn and 80-Mn-NP groups, accompanied by a quadratic effect (p < 0.05). CD was significantly increased (p < 0.05) in birds supplemented with 20-Mn-NP compared with the 40-Mn-NP, 80-Mn-NP, bulk-Mn, and control groups, with both linear and quadratic effects (p < 0.05).

**Table 7:** Effects of Mn-NP supplementation on broiler jejunal microarchitecture.

Parameter	Control	Bulk-Mn (80 mg/kg)	Mn-NP doses (mg/kg)				SEM	p-value	Linear	Quadratic

			20	40	60	80				
VH (µm)	761^ab^	626^b^	923^a^	796^ab^	828^a^	417^c^	32.00	<0.05	0.055	<0.05
VW (µm)	111^ab^	93^b^	121^ab^	138^a^	133^a^	90^b^	5.30	0.021	0.525	0.024
CD (µm)	124^bc^	99^c^	159^a^	115^bc^	135^ab^	51^d^	6.82	<0.05	0.007	<0.05
VH: CD	6.40^b^	6.52^b^	5.79^b^	6.99^ab^	6.40^b^	8.40^a^	0.23	0.035	0.027	0.054
VSA (mm^2^)	0.27^a^	0.18^b^	0.35^a^	0.34^a^	0.34^a^	0.12^b^	0.02	<0.05	0.525	<0.05

Mn-NP = Manganese nanoparticles, VH = Villus height, VW = Villus width, CD = Crystal depth, VSA = Villus surface area, SEM = Standard error of pooled means. ^abc^Means in a row lacking similar superscripts differ significantly at p < 0.05. n = 12 birds/group (3 birds/replicate/group).

The VH-to-CD ratio was significantly higher (p < 0.05) in the 80-Mn-NP group compared with the control, bulk-Mn, 20-Mn-NP, and 60-Mn-NP groups. A significant linear effect (p < 0.05) and a quadratic trend (p = 0.054) were noted. VSA was significantly reduced (p < 0.05) in the 80-Mn-NP and bulk-Mn groups compared with the control, 20-, 40-, and 60-Mn-NP groups, with a strong quadratic effect (p < 0.05).

### Expression of tight junction proteins

**Figure 3:**
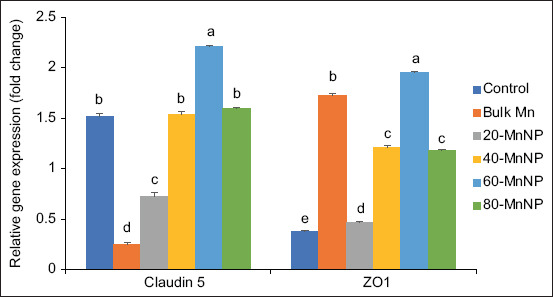
Effects of Mn-NP supplementation on the messenger RNA expression of intestinal genes like zonula occludens-1 (ZO1) and claudin-5 in broilers. Control = basal diet without manganese (Mn) supplementation, Bulk-Mn = Diet supplemented with Mn at 80 mg/kg of diet in the form of MnSO_4_, 20-Mn-NP = 20 mg Mn-NP/kg of diet, 40-Mn-NP = 40 mg Mn-NP/kg of diet, 60-Mn-NP = 60 mg Mn-NP/kg of diet, 80-Mn-NP = 80 mg Mn-NP/kg of diet, Mn-NP = Manganese nanoparticles, SEM = Standard error of pooled means. ^abcde^Mean bars lacking similar superscripts differ significantly at p < 0.05. n = 12 birds/group. Error bars indicate the mean standard error.

As illustrated in Figure 3, the mRNA expression levels of CLDN-5 and ZO-1 were significantly upregulated (p < 0.05) in the 60-Mn-NP group compared with other treatments. Both linear and quadratic effects (p < 0.05) were observed for the expression of these tight junction proteins.

## DISCUSSION

### Growth performance

Mn is an essential trace mineral in poultry nutrition, required for skeletal development, metabolism, immune response, and enzyme activity [34]. In this study, supplementation with 40-Mn-NP improved FCR without affecting BW or FI. Previous research has reported variable effects of Mn supplementation on broiler performance. For instance, supplementation with Mn-loaded chitosan nanoparticles did not significantly alter growth, FI, or FCR compared with micro-Mn sulfate, carbonate, or oxide at dietary levels of 70, 120, and 170 mg/kg, respectively [23]. Similarly, Sunder *et al*. [35] observed no changes in FCR or weight gain when Mn (60, 120, and 240 ppm) was combined with zinc. Ghosh *et al*. [4] also showed that supplementing diets with 50–100 mg/kg Mn had no effect on growth or FI. Comparable findings were reported with MnO and Mn fumarate at 30–240 mg/kg, which did not affect FCR, BW, or mortality [36].

In contrast, Mn-NPs have been associated with improved live weight, weight gain, and FCR compared with bulk Mn [37]. Matuszewski *et al*. [13] further reported that 60% dietary Mn-NP inclusion increased FI and BW without affecting FCR. In yellow-feathered broilers, Mn supplementation enhanced growth during the starter and grower phases, with 120 and 54 mg/kg being optimal, though no effects were observed in the finisher phase [38]. Our findings align with these observations, as Mn-NP supplementation improved FCR during weeks 2 and 3, highlighting the critical role of Mn in the starter period. Differences among studies may reflect variation in species, experimental duration, environmental conditions, or the physical form and dose of Mn used. The enhanced feed efficiency observed with Mn-NP can be attributed to their nanoscale size, improved intestinal absorption, longer retention, and reduced fecal excretion [18–22]. Reduced Mn excretion in Mn-NP-fed birds compared with bulk Mn confirms their higher bioavailability [13], emphasizing their potential for both nutritional and environmental benefits.

### Relative viscera weights

Organ development is essential for supporting growth, preventing defects, and maintaining physiological function [39]. In this study, Mn-NP supplementation did not influence visceral organ development except for the gizzard and cecum. Birds receiving 40-Mn-NP had reduced gizzard weight, whereas bulk-Mn increased cecal weight compared with several treatment groups. These results are consistent with previous reports showing no significant effects of Mn (nano or bulk) at 30%–100% supplementation levels on stomach, liver, or heart weights [13]. Similarly, dietary Mn supplementation (0–140 mg/kg) did not affect spleen, thymus, or bursa weights [38], and inclusion at 30–240 mg/kg had no effect on liver or heart weights in cockerels [36].

### Serum biochemical profile

Serum metabolites serve as important indicators of metabolic health, nutritional status, and disease [40, 41]. In the present study, Mn-NP supplementation did not alter serum biochemical markers, including liver enzymes, kidney function markers, proteins, or lipid profile. This agrees with Matuszewski *et al*. [13], who reported no adverse effects of bulk or NP-MnO supplementation on glucose, AST, ALT, creatinine, TP, LDH, albumin, TG, and TC in broilers. Likewise, Mn amino acid complex supplementation (20–800 mg/kg) did not alter bilirubin, glucose, TP, albumin, uric acid, ALT, AST, or creatinine levels in laying hens [42].

### Meat physicochemical traits

Key physicochemical parameters such as pH, water-holding capacity, and cooking loss influence broiler meat quality, consumer acceptance, and economic returns. In this study, breast muscle pH measured at 45 min postmortem was higher in the 20- and 40-Mn-NP groups but lower in the 80-Mn-NP group. After 24 h, pH declined in the bulk-Mn and 40-Mn-NP groups but increased in the 80-Mn-NP group. Cooking loss and water-holding capacity remained unaffected. Similar findings have been reported, with Mn amino acid complexes increasing early postmortem pH [43], while higher Mn-NP levels reduced pH without affecting WHC or cooking loss [44]. A previous study has also observed no differences between nano and bulk Mn_2O3_ forms at various supplementation levels [12], whereas Mn-NP addition has been shown to increase muscle pH in broilers [45].

Mn plays a role as a cofactor for enzymes such as pyruvate carboxylase and phosphoenolpyruvate carboxykinase [33, 46], which regulate pyruvate metabolism. Under anaerobic conditions, pyruvate is converted to lactic acid, the major cause of postmortem pH decline [47]. This mechanism may explain the observed pH drop in bulk-Mn and 40-Mn-NP groups after 24 h.

Muscle fiber traits are closely linked to growth and meat quality [48, 49]. Birds supplemented with 80-Mn-NP exhibited greater fiber diameter and surface area, while 20-Mn-NP increased fiber density. Larger fibers with a greater cross-sectional area can indicate abnormalities, such as necrosis and degeneration [50], which are associated with rapid muscle growth and increased susceptibility to oxidative stress [51]. Inclusion of metallic nanoparticles in broiler diets has also been reported to increase fiber size and reduce fiber number [52]. However, the effects of Mn-NPs on muscle histology remain underexplored.

### Jejunal morphology

Jejunal histology reflects digestive and absorptive capacity [53]. Villi arise from crypt regions containing stem cells, and deeper crypts produce taller villi [54]. In this study, 60-Mn-NP supplementation increased VH and width, while 20-Mn-NP increased CD. Birds receiving 80-Mn-NP showed an increased VH-to-CD ratio but reduced VSA, suggesting high doses may suppress villus growth. Similar findings were reported by Zhang *et al*. [5], who found Mn supplementation (0–100 mg/kg) increased duodenal VH but had no effect on jejunal parameters. The enhanced villus morphology at 60-Mn-NP suggests improved intestinal health and absorptive function.

### Expression of tight junction proteins

Mn supports intestinal barrier integrity by regulating tight junction proteins. Mn deficiency reduces tight junction expression and compromises intestinal permeability [5]. In this study, supplementation with 60-Mn-NP significantly upregulated CLDN-5 and ZO-1 expression. CLDN-5, a tight junction component, regulates paracellular transport and contributes to intestinal epithelial differentiation [55]. Our results align with prior work showing that Mn intake enhances tight junction expression, while deficiency decreases ZO-1 and CLDN-1 expression during S. typhimurium infection [5]. The upregulation observed at 60-Mn-NP may reflect an optimal dose supporting enzyme activity and antioxidant defense, whereas higher levels could disrupt mineral balance, trigger oxidative stress, or induce feedback regulation, limiting protein expression.

### Practical and environmental implications

Supplementation with Mn-NPs offers multiple benefits: improved bioavailability, reduced fecal excretion, and minimized environmental contamination compared with conventional Mn sources. Although Mn-NP synthesis is currently more expensive than bulk Mn, its superior efficiency allows for lower dietary inclusion, potentially reducing overall mineral use, feed costs, and waste. With advances in nanotechnology, large-scale production is expected to become more economical, enabling Mn-NP to serve as a sustainable strategy for enhancing poultry performance while mitigating environmental impact.

## CONCLUSION

This study demonstrated that dietary supplementation with Mn-NPs exerted beneficial effects on broiler performance, intestinal health, and muscle characteristics without inducing systemic toxicity. Growth performance was not adversely affected, and improvements in FCR were evident, particularly with 40-Mn-NP supplementation during the starter phase. Relative visceral organ weights remained largely unaffected, except for reduced gizzard weight at 40-Mn-NP and increased cecal weight in the bulk-Mn group. Serum biochemical parameters, including liver and kidney markers, lipid profile, and protein levels, remained stable across all treatments, confirming the safety of Mn-NP supplementation.

From a meat quality perspective, Mn-NP influenced early and late postmortem muscle pH, while cooking loss and water-holding capacity were unaffected. Birds supplemented with 80-Mn-NP showed enlarged muscle fibers, whereas 20-Mn-NP increased fiber density, indicating differential effects of dosage on muscle histomorphometry. Jejunal morphology was improved with 60-Mn-NP, which enhanced VH, width, and surface area, while 20-Mn-NP deepened crypts and 80-Mn-NP reduced surface area. At the molecular level, 60-Mn-NP significantly upregulated the expression of tight junction proteins (CLDN-5 and ZO-1), highlighting its role in maintaining intestinal barrier integrity.

The strength of this study lies in its comprehensive evaluation of Mn-NP effects, spanning growth, visceral organs, serum biochemistry, meat traits, histomorphometry, intestinal morphology, and gene expression. Collectively, these findings support the potential of Mn-NP as a sustainable alternative to conventional Mn supplementation, as it improves bioavailability, reduces excretion, and minimizes environmental contamination.

However, limitations include the relatively short trial duration and the absence of long-term safety assessments such as cumulative Mn deposition in tissues and oxidative stress biomarkers. Additionally, the cost of Mn-NP synthesis remains a practical barrier, although advances in nanotechnology may reduce expenses for commercial applications.

Future research should focus on long-term feeding trials to assess safety and Mn tissue deposition, the interaction of Mn-NP with other trace minerals, and its effects under commercial farming conditions. Investigating the influence of Mn-NP on gut microbiota, oxidative stress regulation, and immune responses would also provide deeper mechanistic insights.

In conclusion, Mn-NP supplementation at 40–60 mg/kg optimally improved feed efficiency, intestinal morphology, and barrier function without compromising systemic health. These findings provide a strong foundation for incorporating Mn-NP into poultry nutrition strategies, thereby contributing to enhanced performance, improved meat quality, and increased environmental sustainability.

## AUTHORS’ CONTRIBUTIONS

MS, HR, SKT, and MSY: Conceptualization. MS, HZ, MN, and SKT: Sampling and analysis. MS, HR, SKT, MSY, HZ, and MN: Data analyses. MS: Writing – original draft preparation. SKT, MSY, HZ, MN, and HR: Writing – review and editing. SKT and HR: Supervision. All authors have read and approved the final version of the manuscript.
